# Bayesian Variable Selection in Searching for Additive and Dominant Effects in Genome-Wide Data

**DOI:** 10.1371/journal.pone.0029115

**Published:** 2012-01-03

**Authors:** Tomi Peltola, Pekka Marttinen, Antti Jula, Veikko Salomaa, Markus Perola, Aki Vehtari

**Affiliations:** 1 Department of Biomedical Engineering and Computational Science, Aalto University School of Science, Espoo, Finland; 2 Department of Health and Functional Capacity, National Institute for Health and Welfare, Turku, Finland; 3 Department of Chronic Disease Prevention, National Institute for Health and Welfare, Helsinki, Finland; 4 Public Health Genomics Unit, Department of Chronic Disease Prevention, National Institute for Health and Welfare, Helsinki, Finland; 5 Institute for Molecular Medicine Finland (FIMM), University of Helsinki, Helsinki, Finland; 6 Estonian Genome Center, University of Tartu, Tartu, Estonia; Aarhus University, Denmark

## Abstract

Although complex diseases and traits are thought to have multifactorial genetic basis, the common methods in genome-wide association analyses test each variant for association independent of the others. This computational simplification may lead to reduced power to identify variants with small effect sizes and requires correcting for multiple hypothesis tests with complex relationships. However, advances in computational methods and increase in computational resources are enabling the computation of models that adhere more closely to the theory of multifactorial inheritance. Here, a Bayesian variable selection and model averaging approach is formulated for searching for additive and dominant genetic effects. The approach considers simultaneously all available variants for inclusion as predictors in a linear genotype-phenotype mapping and averages over the uncertainty in the variable selection. This leads to naturally interpretable summary quantities on the significances of the variants and their contribution to the genetic basis of the studied trait. We first characterize the behavior of the approach in simulations. The results indicate a gain in the causal variant identification performance when additive and dominant variation are simulated, with a negligible loss of power in purely additive case. An application to the analysis of high- and low-density lipoprotein cholesterol levels in a dataset of 3895 Finns is then presented, demonstrating the feasibility of the approach at the current scale of single-nucleotide polymorphism data. We describe a Markov chain Monte Carlo algorithm for the computation and give suggestions on the specification of prior parameters using commonly available prior information. An open-source software implementing the method is available at http://www.lce.hut.fi/research/mm/bmagwa/ and https://github.com/to-mi/.

## Introduction

In recent years, numerous genome-wide association studies (GWAS) have been successful in locating disease or trait associated variations in the human genome (see, e.g., discussion by Lander [1]). The analyses are usually conducted by interrogating the effect of a single genetic variant at a time and setting a stringent threshold for statistical significance to account for multiple hypothesis testing. While computationally convenient with the hundreds of thousands of variants often genotyped, the strategy is suboptimal, leaving just below the statistical significance a “gray area” of variants. The identified variants often account only for a minor portion of the estimated heritability of complex traits [2].

Advances in approximate computation and the increasing computational resources have facilitated the computation of models that simultaneously consider all variants, with demonstratively better performance for identifying trait associated variants at least in simulations [3]–[6]. These methods adhere more closely to the hypothesis of multiple variants affecting complex traits and gain power from accounting for the multiple genetic effects simultaneously. Moreover, some formulations of the problem, specifically Bayesian variable selection and model averaging (BMA), naturally provide estimates of the uncertainties in the quantities of interest and allow for inferences beyond the marginal significance of single variants. For example, Wilson et al. [5] compute probabilities of association for regions of the genome (e.g., genes), and Guan and Stephens [6] estimate the heritabilities of traits (to the extent explained by the available genetic data). The flexibility of the approach also allows for extensions to simultaneous analysis of multiple traits [7], [8] and interactions [9], [10].

Here, we study the potential of BMA in genome-wide modeling of additive and dominant genetic variation. Although, in principle, a simple extension of the additive genetic model, it is computationally burdensome and can lead to a reduction in the power of identifying associated loci. We focus our analysis on a formulation of genetic effects, where each variant can contribute either an additive or an additive and a heterozygosity term. This formulation allows for the modeling of (complete and incomplete) dominance. As baseline models, we use a purely additive formulation and a pseudo-SNP approach, where the number of variants is naively doubled by introducing an additional pseudo variant with heterozygous coding for each genetic variant. Rationale for the latter is that it allows for the modeling of dominance and any software handling the basic additive formulation could be used for the analysis. The behavior of these models is studied in simulations based on real single-nucleotide polymorphism (SNP) data and the results are compared against conventional single-SNP analysis. An application to the analysis of the genetic basis of variation in key lipid metabolism components in the circulation, high-density (HDL-C) and low-density lipoprotein cholesterol (LDL-C), is then presented. The dataset consists of 3895 Finnish individuals with (imputed) genotypes available on over one million SNPs.

The simultaneous identification of trait associated (causal or correlated to causal) variants is facilitated by modeling the genotype-phenotype mapping as a sparse multiple linear regression, where only a small proportion of the variants is expected to have non-zero effects. Specifically, our formulation corresponds to a type of spike-and-slab prior [11], [12], which is a popular choice in Bayesian variable selection with a large number of variables. In the context of GWAS, this allows us to naturally incorporate prior knowledge on the expected number of associated loci and effect sizes or heritability, which are nowadays often available from previous studies for common traits. To learn which of the hundreds of thousands or millions of variants have non-zero effects (i.e., are associated to the trait) is a great computational challenge. To this end, such a prior structure for the linear model is assumed, which allows analytic integration over several of its parameters. Sampling is then utilized to identify the associated variants. Specifically, we present a Markov chain Monte Carlo (MCMC) algorithm, which samples from posterior distribution of the model space by proposing additions, removals and swaps of the variants that are allowed to have non-zero effects. The sampling effort is focused more on variants showing some effect on the trait by adapting the proposal distribution of additions to the marginal association probabilities during an initial phase of the sampling. Sampling algorithms with similar rationales have been utilized at least by Clyde et al. [13], Guan and Stephens [6] and Nott and Kohn [14].

An excellent general discussion on Bayesian variable selection in GWAS is provided by Guan and Stephens [6]. Our primary contribution here is to investigate extending this approach to simultaneously model both additive and dominant genetic effects. Other authors (e.g., [5], [9], [15]) have previously studied BMA models, which include terms for dominance variation, but they have not explicitly focused on this and their prior for the effect types have been different and the scale of datasets smaller. Our formulation seems robust with regard to the potential loss of power in such extensions of model space, and may lead to improved estimates of key quantities such as the heritability of a trait. These results may be seen as demonstrations of the benefits of explicitly accounting for the multifactorial genetic basis of complex traits within hierarchical modeling.

## Methods

### Model

Let 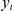, 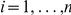, be measurements of a continuous phenotype of interest for 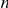 individuals, and 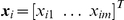, 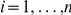, be vectors of the values for 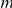 (usually 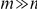) genetic variants (here, the numbers of minor alleles of SNPs) for each individual. The trait is then modeled as a linear combination of the variants and other covariates 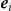:
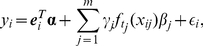
(1)where 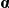 and 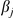 are the regression coefficients and 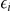 is the residual. The binary variables 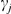 indicate which effects are included in the model. The function 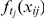 describes the type of the effect of SNP 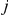. In the case of additive genetic model, this is 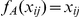.

#### Types of genetic effects

Let M and m be the major and minor alleles at a SNP. The following types of genetic effects may be considered:

- Additive (A) with 0, 1, 2 coding for the genotypes MM, Mm/mM, mm.
- Heterozygous (or dominance deviation; H) with 0, 1, 0 coding for MM, Mm/mM, mm.
- Dominant (with respect to the minor allele; D) with 0, 1, 1 coding for MM, Mm/mM, mm.
- Recessive (with respect to the minor allele; R) with 0, 0, 1 coding for MM, Mm/mM, mm.

The model indicator 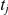 obtains values in 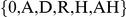 with the functions 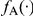, 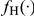, 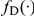, 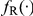 formed according to the above codings and 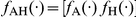 (a two-element vector of the A and H functions). The effect type 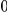 is fixed for 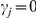, where the coding does not matter.

The attention will be restricted to models, which allow 1) additive effects (referred to as BMA A), 2) additive or additive and heterozygous effects (BMA A/AH) and 3) additive effects with pseudo variants (BMA pseudo); see Table 1. The first model is commonly used in genome-wide association studies, but it does not model dominance. The second model is our primary interest. Note that the fully dominant (D) and recessive (R) effects are special cases of AH with 
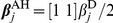
 and 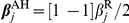, respectively. The third model is handled identically to BMA A, but the number of variants is doubled by adding a pseudo variant with heterozygous coding per each original variant.

**Table 1:** Bayesian model averaging formulations and genetic effects.

Model	Allowed effects	Model space size
BMA A	0, A	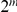
BMA A/AH	0, A, AH	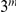
BMA pseudo	0, A	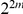

Model space refers to the possible combinations of 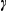 and 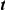. 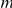 is the number of variants. Pseudo refers to including a pseudo-SNP with heterozygosity coding for each SNP.

Table 1 lists the model space sizes. The model spaces are enlarged by factors of 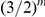 and 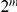 for BMA A/AH and pseudo, respectively, compared to BMA A.

#### Model space prior

The variable selection is facilitated by placing a prior distribution on the vector 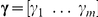, which controls the inclusion of variants into the model. A common choice is (with the notation for probability distributions following Gelman et al. [16])
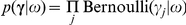


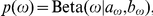
where 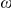 is the probability of including a variant in the model and can be integrated out analytically. The prior serves a similar role to the classical multiple hypothesis testing correction [17]. Kohn et al. [18] give formulae for determining the hyperparameters 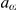 and 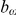 by considering the expected number of associated variables and its variance. This is natural also in the present application as there often are available some broad estimates of the number of causal variants from previous studies.

A functionally similar prior can be used for the effect types 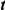 given 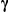:
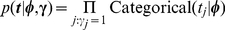
(2)

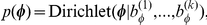
where 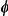 is a vector of the probabilities of the different genetic effects. This also allows integrating 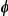 out analytically and provides a further multiple testing adjustment. The parameters 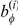 can be thought of as prior samples of the different types of effects. For example, setting them to large equal values would effectively correspond to giving probability 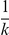 for each effect type.

#### Priors of the linear model

In matrix notation, the regression model in Equation 1 can be written as
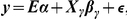
where 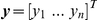, 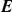 is the design matrix of fixed covariates, 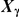 is the design matrix of the variants for which 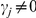 formed according to the above discussion of effect types, 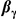 is the vector of the corresponding regression coefficients and 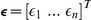.

The distribution of the residual is assumed normal
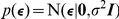
and conjugate prior distributions are placed on 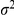, 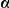 and 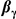, with 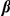 following a spike-and-slab formulation [11], [12]:
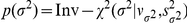


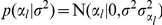


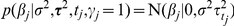


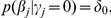
where 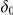 is the Dirac delta function at zero (the inclusion of 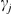
s in the Equation 1 was redundant). Note that 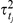 are allowed to be different depending on the effect type (e.g., for BMA A/AH we have 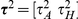; here, for a variant with 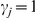 and 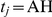, 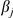 has actually two components with prior variances 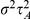 and 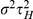). The structure allows marginalizing over 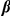 and 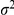 analytically given the other parameters. A popular alternative prior for 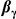 is the g-prior [19]. However, some of its properties seem undesirable for application in GWAS: in particular, the assumption about the correlation structure of the genetic effects (see [6]) and the implied smaller shrinkage for the effects of rare variants (see [20] for a non-GWAS specific discussion).

The prior for the variable selection coefficients are given the semi-conjugate form
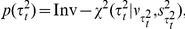
which allows convenient sampling of 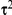 given the other parameters. Guan and Stephens [6] use an alternative formulation, where the prior for 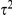 is indirectly induced through a prior on heritability and depends on 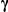. We use similar reasoning to guide the specification of hyperparameters (see below), but do not explicitly tie 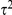 and 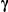. The question whether the parameters should be tied in the prior relates to whether one is more confident in specifying prior information on the effect sizes or on how much of the phenotypic variance the available genetic data could overall explain.

Finally, to account for missing data a categorical prior is placed on an element of 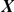:
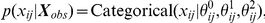
where 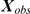 refers to the observed genotype data and 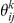 is the prior probability of the genotype 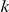. Thus, for observed data with genotype 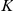, 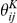 is set to one and other 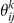 to zero and for missing data, 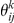
s are set to the marginal distribution of the genotypes for the corresponding SNP. In general, the 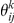
s could be set, for example, to the genotype probabilities from imputation algorithms.

#### Elicitation of 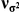, 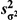, 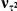 and 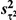


Often estimates of the heritability of the trait are available from published genome-wide association studies or from some other sources (of course, often the studied data cannot be assumed to exhaustively cover all genetic variation, but is restricted to, for example, SNPs). This prior knowledge can be used to guide the setting of the hyperparameters of the variance distributions in the model.

First, note that the proportion of variance explained by the linear model
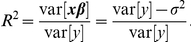
Placing a distribution with density 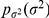 (here 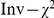) on 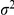 induces a distribution for 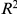, which given the population variance 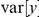 has density 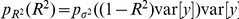. We suggest setting the hyperparameters 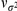 and 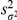 by inspecting the implied prior on 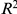. While the prior can have mass on negative values of 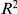, the likelihood of the linear model will usually be concentrated on the positive values or around zero if there is no explanatory power in the model. The connection to heritability estimates can be made by assuming independent contributions of genetic (heritability 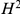) and environmental (known covariates; 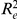) effects. Then 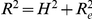 and, for example, an empirical estimate of 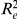 from simple linear regression may be used. One possibility is then to fix the mode of prior to the expected 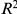 and choose the other degree of freedom so that the prior is relatively flat around plausible values of 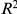.

Similar derivation can be made for a single effect with heritability contribution 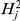:
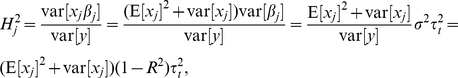
where 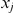 and 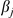 are assumed independent. Now, given 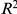, 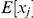 and 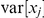 setting a distribution for 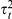 induces a distribution for 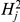. To solve for the hyperparameters 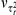 and 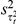, one can consider setting 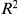 to its expected value and set the expectation and variance of 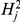 according to prior knowledge (e.g., coarsely setting 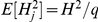 with 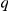 being the expected number of causal variants). The support of the prior may then be checked over a range of plausible 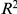 values. We have used the sample estimates of 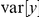, 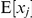 and 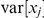 (specifically, mean over all variants and mean of the variances of the variants) in our experiments.

#### Covariates 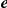


A constant term of ones is included to account for non-zero mean of the trait. An improper prior is placed for the corresponding regression coefficient: 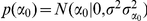, where 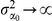. While this makes the marginal likelihood of the linear model (Text S1, step 3) tend to zero, the posterior distribution of 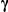 and the Bayes factors required in the computation have proper limits (see Protocol S1 in the supplementary materials of [15]). For other fixed covariates, the variance parameters 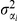 are set to suitable (fixed) values.

### Computation

Markov chain Monte Carlo (MCMC) sampling is used to generate samples from the posterior distribution of the model parameters. The sampling of 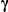 and 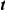 is performed with Metropolis-Hastings algorithm [21], [22], where local updates are proposed as explained below. Gibbs sampling [23] scheme is used to update the parameters sequentially. The sampling consists of iterating five steps (see Text S1 for brief derivations of the sampling distributions):

1. Sample 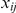 given 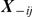, 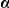, 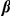, 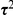, 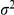, 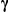, 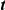, 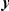 for all missing data from categorical distributions. Sampling of missing genotypes in variants that are not included in the model (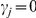) can be disregarded as they do not affect the sampling of the other parameters. An exception is such a variant that is considered for addition to the model in the third step.
2. Sample 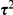 given 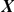, 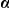, 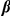, 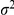, 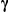, 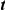, 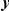 from (independent) 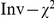 distributions.
3. Sample 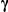, 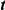 given 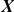, 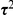, 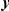 by a local Metropolis-Hastings move (see below). Note that 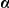, 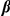 and 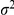 can be integrated out analytically at this step.
4. Sample 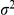 given 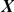, 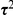, 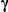, 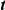, 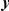 from 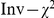 distribution.
5. Sample 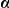, 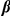 given 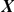, 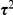, 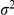, 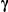, 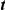, 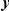 from multivariate normal distribution.

Note that the steps 3–5 can be seen as a draw from a block distribution 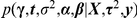, which is decomposed into three steps. Steps 1 and 2 sample from full conditionals. While the local moves at step 3 require only updates of complexity 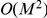 to the Cholesky decomposition used in the regression, step 1 requires the computation of a full Cholesky decomposition of complexity 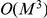, where 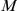 is the number of variables with 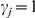. As 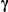 is of primary interest and the local updates lead to large autocorrelations, step 3 is repeated a number of times (here 10) before moving on.

#### Local updates to 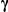, 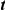


After proposing an update of 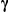, 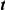 the move is accepted or rejected according to the Metropolis-Hastings acceptance probability 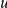:
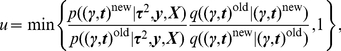
(3)where 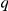 is the proposal distribution, which is here decomposed into three steps: 1) update type, 2) variant and 3) effect type.

Four types of updates are considered:

1. addition of a variant to the model,
2. removal of a variant from the model,
3. switch of two variants (combination of the two above),
4. switch of effect type for a variant,

with probabilities 0.4, 0.4, 0.1 and 0.1. For updates 2 and 4, the variant is chosen randomly among the variants in the model (disregarding the effect type). Update 3 is formed as a composition of updates 1 and 2. The proposal distributions for additions and selection of effect types are formed adaptively during an initial sampling period as explained below, after which they are fixed. Samples from the adaptive phase are not used for posterior inference.

The proposal distribution for additions is formed according to the marginal association probabilities of the variants (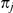), which are updated during an initial sampling phase. The values are initialized to the single-SNP association probabilities and updated according to the Rao-Blackwellized probabilities (see below; computed every 1000th iteration). Specifically, the proposal probability for addition of 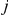
th variant is 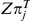, where 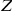 normalizes the distribution over all 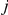 and 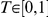 is a prespecified constant, which can be used to flatten the distribution (e.g., 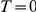 leads to uniform proposals, we have used 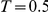). The proposal distributions for the effect types are formed similarly, but the probabilities for effect types are calculated for each variant independently. The rationale of this proposal strategy is to guide the sampling to those SNPs that are more likely to be significant and thus, to increase the acceptance and convergence rates of the sampler in cases with a very large number of variables, with most variables expected to be insignificant.

#### Posterior association probabilities

Guan and Stephens [6] propose using Rao-Blackwellized estimates of posterior association probabilities to reduce sampling variance relative to MCMC frequency estimates. Following their derivations, the estimate of the marginal association probability is computed as a mean over the MCMC samples:
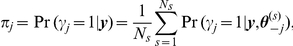
where 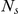 is the number of samples and 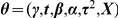 (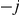 indicates the removal of parameters specific to variable 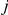). Here, the probabilities of effect types (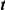) are also tracked by computing 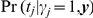 in a similar fashion. Both of these can be computed by computing the odds
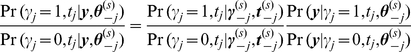
for each value of 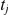. The Bayes factors are fast to compute as they are linear regressions of two or three variables (constant and one or two terms for the variant) [6].

Single-SNP analysis and the Bayesian model averaging (BMA) approach behave differently in the estimation of the marginal significances of variants in regions with high linkage disequilibrium (LD): while single-SNP methods report similar significances for the variants in the region, BMA tends to dilute the posterior probability mass among the variants, since only one of them is needed in the model. It is then sensible to compute the posterior probabilities for regions of the genome. Often the actual causal variants cannot anyway be assumed to be among those genotyped. Unfortunately, the Rao-Blackwellization approach is not feasible for this, although summing over the association probabilities within a region can be used as an estimate of the number of associated variants within the region [6]. However, the association probabilities over large regions could be expected to suffer less from sampling variance than over single variants. Thus, frequency estimates from the MCMC chains are used instead. With 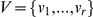 defining the indices of the variants within the region
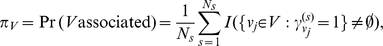
where 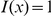 if 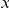 is true and 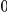 otherwise. Note that the prior probability of association of 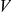 depends on the size 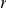 of the region. For moderate sizes the prior probabilities are small. It is also possible to compute Bayes factors comparing the hypothesis of association of a region to no association [5]. The probabilities of effect types for a region are computed by frequency estimates over MCMC samples, in which at least one variant within the region is included in the model.

### Ethics statement

Human data was not collected primarily for this article and was analyzed here anonymously. Primary collection has followed appropriate ethics guidelines.

## Results

### Simulations

Simulations were used to characterize the behavior of the models and to validate the approach against a single-SNP approach implemented in the popular software PLINK (version 1.07; see Text S2 for the used analysis options) [24]. To account for the linkage disequilibrium structure of the genome, real genotype data of chromosome 1 from 2002 individuals was used in the simulations. This consisted of 85,331 SNPs after imputation and quality control. Quantitative traits were simulated according to a linear model with the following steps: 30 causal variants were selected randomly among the SNPs, effect types were either all set to additive (sim A) or selected randomly from additive, dominant or recessive (sim A/D/R). The effect sizes were generated from a double exponential distribution and normally distributed noise was added to achieve a preset level of heritability 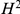 (0.2 or 0.5). Forty datasets were simulated with each parameter configuration.

Weakly informative prior distributions were used for the Bayesian model averaging (BMA). Specifically, correct values were used to set means and modes, but the distributions were given large variances (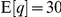, 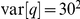, where 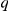 is the number of included variants, 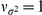, mode of 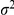 was set according to 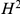, 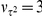, 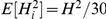, 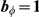). Two chains of length two million samples were run for each dataset and model. Second halves of the chains were thinned by taking every 100th sample and used for posterior inference.

#### Causal variant identification performance

To make BMA and PLINK better comparable, the genetic map of chromosome 1 from HapMap (phase 2, release 22) [25] was used to divide the SNPs into regions with boundaries at loci, where adjacent SNPs were more than 0.01 cM apart. This resulted into 3776 regions. Similar results were obtained by using a lower threshold (0.005 cM; 6421 regions; Figure S1) or by dividing the SNPs into LD blocks with an algorithm in Haploview software [26] (Figure S2). For single-SNP results, minimum of the p-values within a region was taken. For BMA, region-wise marginal posterior probabilities were computed as frequency estimates from the MCMC chains.

**Figure 1:**
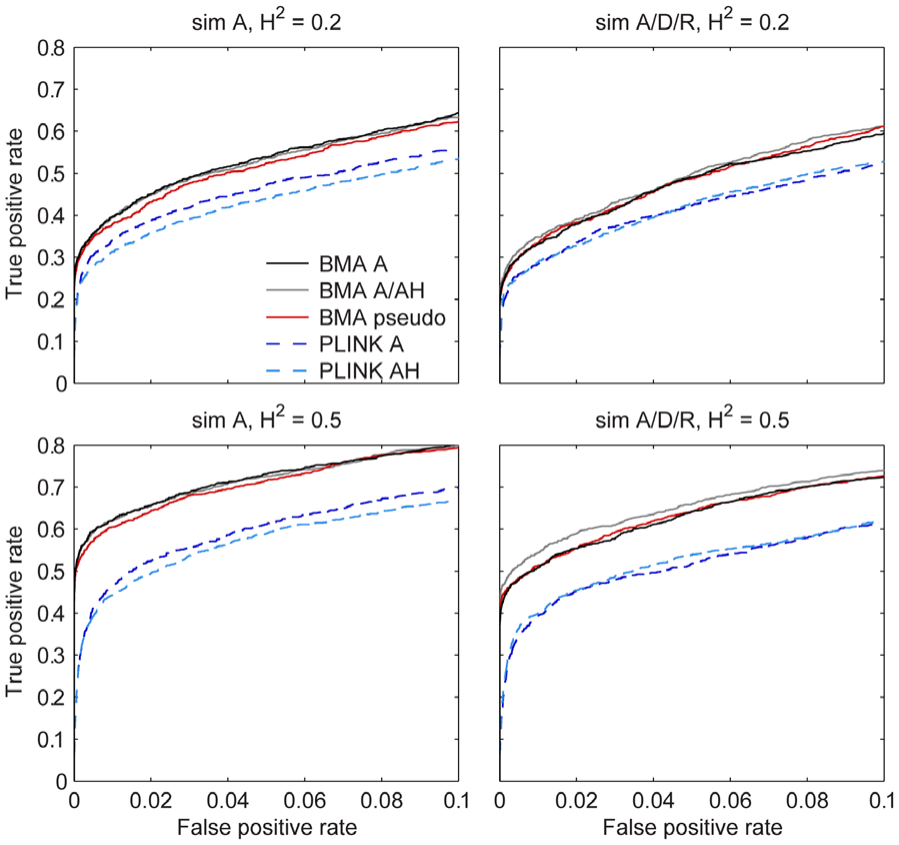
Causal variant identification performance in simulations. True positive rate as a function of false positive rate in simulations with all forty replicate datasets combined within each configuration (i.e., each dataset has the same cutoff for calling positives and the number of true and false positives are summed over the datasets).

Figure 1 shows the true positive rates as a function of the false positive rates for the approaches, with the forty replicate simulations combined (similar to Guan and Stephens [6]). BMA has clearly better performance in these simulations than conventional single-SNP analysis (PLINK and single-SNP posterior probabilities gave similar results; Figure S3). The difference is larger with the higher heritability. This is plausible as there is less residual variation available for producing chance associations in the multivariate linear models after the strongest associations have been accounted for. When only additive effects (sim A) are simulated, there is little difference between BMA A and BMA A/AH, but BMA pseudo performs slightly worse. Single-SNP analysis with AH model suffers a small drop in performance compared to only A. With additive, dominant and recessive effects (sim A/D/R) BMA A/AH shows some improvement over BMA A and pseudo. The improvement is larger with higher heritability.

#### Effect type identification

Effect type identification accuracies for causal variants were computed on the region level weighted with posterior association probabilities 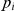:
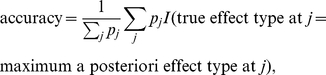
(4)where 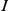 is an indicator function with value 1 if the argument is true and 0 otherwise. Simulated D and R effects were classified as AH. The effect type of a SNP in BMA pseudo was classified as AH if the pseudo term was present in the model and A otherwise. The motivation for the weighting scheme is that only the effect types of variants with high posterior association probability are of interest.

Table 2 shows the accuracies as an average over the replicate simulations. The results are mixed. Both BMA A/AH and pseudo have high accuracies, when only additive effects are simulated, especially with the higher heritability and clearly have some ability to identify dominant and recessive effects, although the accuracies are well below 80% even in the higher heritability simulations. Looking more closely at the types of errors of BMA A/AH, there is little difference in percentages of confusing A to AH and AH to A (both are 30% with 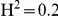 and 25% with 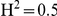).

**Table 2:** Effect type identification accuracy in simulations (weighted with posterior association probability).

sim. effects	sim. 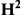	BMA A/AH	BMA pseudo
sim A	0.2	86%	94%
sim A/D/R	0.2	70%	66%
sim A	0.5	98%	97%
sim A/D/R	0.5	75%	76%

#### Heritability and model sizes

**Figure 2:**
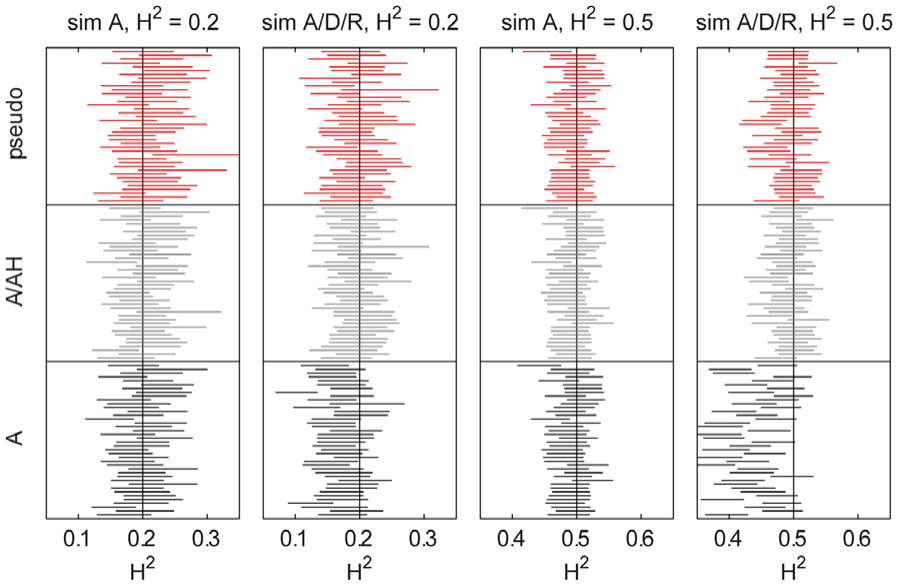
BMA 95% central posterior intervals for heritability in simulations. Each of the forty replicate datasets within all configurations are shown for BMA A, A/AH and A/D/R. The true value is indicated with a vertical line.

The Bayesian model averaging approach facilitates inferences on heritability (calculated as the proportion of variance explained by the genetic effects from the MCMC samples) and the number of causal variants. Figure 2 shows 95% central posterior intervals for heritability in the simulated datasets. With only additive effects, all BMA formulations cover the true value in most replicate simulations. Lower heritability leads to longer intervals as there is generally more uncertainty about the model parameters. With additive, dominant and recessive effects (sim A/D/R), BMA A is biased to lower than true values, especially with the higher heritability, while BMA A/AH and pseudo have good coverage of the true values.

**Figure 3:**
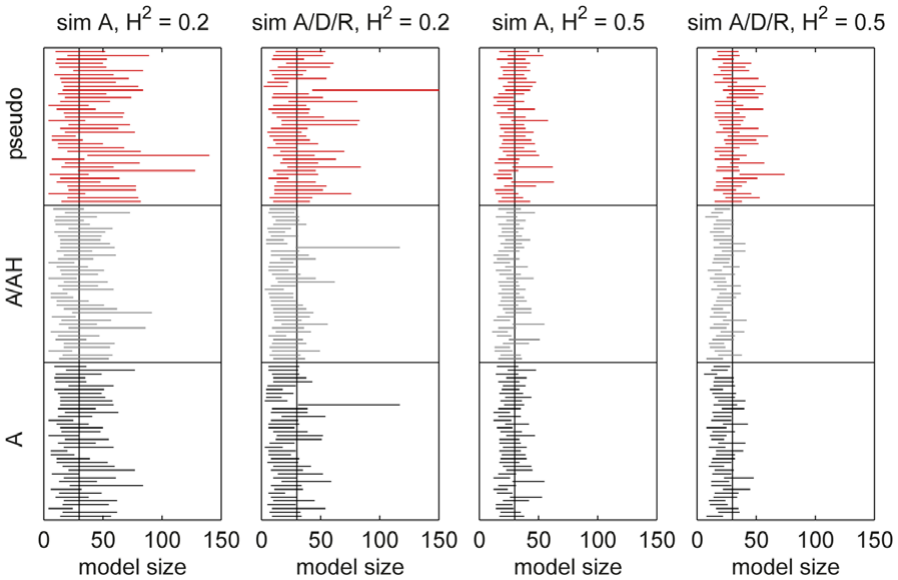
BMA 95% central posterior intervals for the number of causal variants in simulations. Each of the forty replicate datasets within all configurations are shown for BMA A, A/AH and A/D/R. The true value is indicated with a vertical line.

The model size distributions are summarized with 95% central posterior intervals in Figure 3. Only distinct SNPs are counted in BMA pseudo (i.e., having both A and H terms of the same SNP count only as one). Three trends are visible. First, lower heritability leads to larger uncertainty in the number of variants to include. Second, the model sizes are generally biased to smaller than true values. This may be explained by the discrepancy in the prior (normal) and the simulation (double exponential) distributions for the effect sizes. It is probable that many of the simulated effects are small, falling below the implicit identification threshold, and do not contribute much to heritability. Third, BMA pseudo produces on average larger models than BMA A or A/AH and has wider posterior intervals.

### HDL-C and LDL-C analysis

The Bayesian model averaging (BMA) approach was then applied to real data. BMA A (additive) and A/AH (additive or additive and heterozygous terms) models were used.

#### Data and prior parameters

Data were available from 3895 Finnish individuals from two studies. 2002 of the individuals were from a metabolic syndrome case-control sample [27] and 1893 were controls of another study (a subgroup of FINRISK study [28]). Data on high-density lipoprotein blood cholesterol (HDL-C) levels was available for all individuals and on low-density lipoprotein cholesterol (LDL-C) for 3822 individuals. LDL-C values were estimated with the Friedewald formula [29]. The genotype data were imputed with IMPUTE2 program using HapMap 3 reference samples with an additional Finnish founder population reference [30]. Maximum a posteriori genotypes were used from the imputation, which allows for simple handling of multiple effect types with memory-efficient implementation (posterior mean genotypes have been recommended as an approximation to sampling over the imputation uncertainty, see [31]). Missing values in the genotyped SNPs were not imputed. After imputation, 1,051,811 SNPs passed quality control (imputation certainty 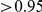, Hardy-Weinberg equilibrium 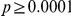, minor allele frequency 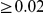 and missingness 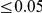; adjacent SNPs with identical genotypes were removed) and were available for both datasets. Study indicator, metabolic syndrome case-control indicator, age, 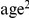, sex, body-mass index, lipid lowering medication together with coarse indicators from questionnaires on education, physical activity and alcohol use were included as covariates. Missing covariates were imputed with regression models based on the other covariates using mi-package in R [32]. Ten principal components with the largest eigenvalues were estimated [33] from the genotyped SNPs (i.e., excluding imputed) and included as covariates to account for population stratification.

Location parameters of the prior distributions were specified based on a recently published large meta-analysis investigating blood concentrations of lipids [34]. Prior variances were set to relatively large. For HDL-C: 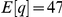, which is the number of identified SNPs in the meta-analysis, 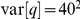, 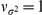, mode of 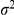 was set according to heritability estimate from the meta-analysis 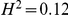 and empirical 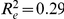 for the covariates, 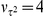, 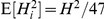, 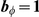. For LDL-C: 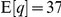, 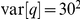, 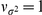, 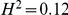 and empirical 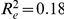 for the covariates, 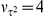, 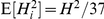, 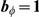. Three MCMC chains of length six million iterations were run for each model and dataset and thinned by taking every 100th sample. Only the second halves were used for posterior inference. Convergence was assessed by visually comparing the three chains and by computing the potential scale reduction factor [16] for shared continuous parameters. These did not indicate any problems. Comparing the marginal posterior association probabilities of SNPs between chains shows some problems in mixing between correlated variants (Figure S4). However, region-wise probabilities do not seem to suffer from this (Figure S4).

#### Posterior association probabilities

**Figure 4:**
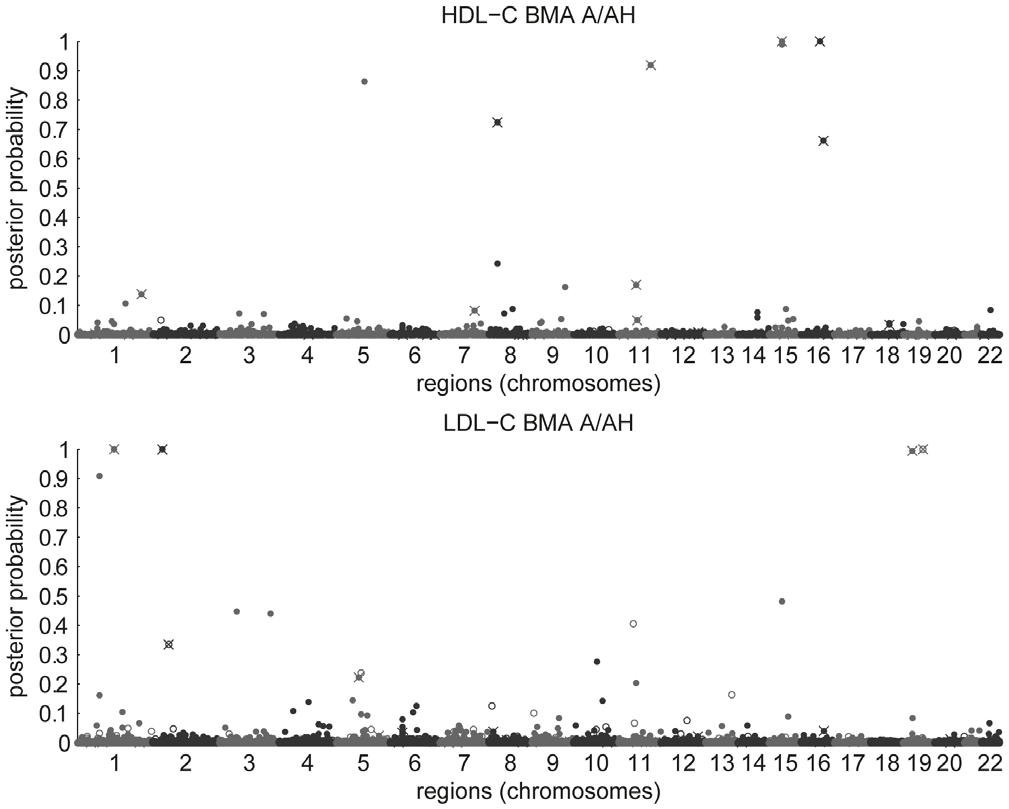
BMA A/AH posterior association probabilities for HDL-C and LDL-C. The SNPs have been divided into 46,172 regions based on HapMap genetic map. Regions, where AH effect has higher probability than A are shown as hollow circles, others as filled. Crosses indicate loci identified in a large meta-analysis [34]. Two close-by regions on chromosome 15 have probabilities near one for HDL-C, but only one can be seen due to overlapping markers.

**Figure 5:**
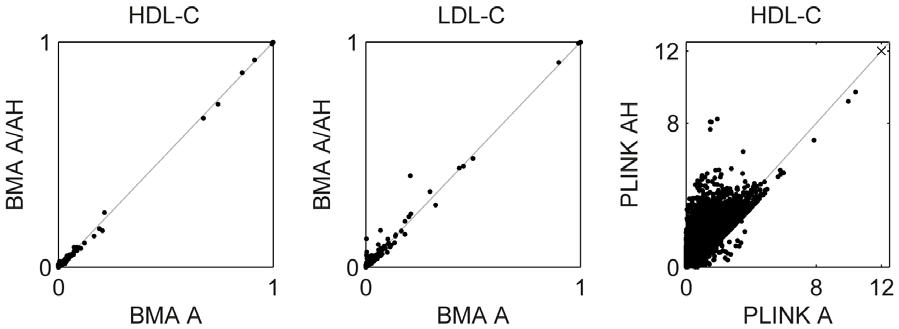
Comparison of region-wise posterior association probabilities for BMA A and A/AH. Similar plot for PLINK 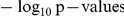 is included for reference (region-wise maximum values; one point with values 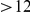 is shown with a cross).

The posterior association probabilities were computed for regions of the genome using HapMap genetic maps (phase 2, release 22) [25]. The variants were assigned into regions with boundaries at loci, where adjacent SNPs in the genetic map were more than 0.01 cM apart. The posterior probabilities for the resulting 46,172 regions are shown in Figure 4 for BMA A/AH. BMA A gives very similar results for both traits (Figure 5) and is not shown. Ten randomly permuted versions of the HDL-C dataset were analyzed for reference, the results of which show no region-wise posterior association probabilities over 0.5 (Figure S5).

For HDL-C, there are seven regions with posterior association probability over half, five of which have been identified previously [34]. Computing estimates for the number of associated loci in each region indicates that the region with probability near one in chromosome 16 harbors two associations (Figure S6). The SNPs showing association in this region are located immediately upstream or in the *CETP* gene. The two associations (one of which is in region not reported by Teslovich [34]) in chromosome 15 are in or upstream of *LIPC* gene. The previously unreported (to our knowledge) putative association on chromosome 5 is about 14 kbp upstream of *CAST* gene and is suspect to being a false positive.

For LDL-C, there are five regions with posterior association probability over half, four of which are in regions, where LDL-C associated SNPs have been found previously in the large meta-analysis study [34]. Closer inspection shows that the fifth association, in chromosome 1, is located in gene *PCSK9*, in and near which associations to LDL-C have also been previously reported. The estimate for the number of associations in this region is 1.6 (Figure S6) with some weaker signals in the near-by *USP24* gene. The second associated region in chromosome 19 has an estimated number of associations of 1.4. This region is located around *TOMM40*, *APOE* and *APOC1* genes, variants in which have been previously found associated to cholesterol levels (the latter two code for apolipoproteins).

Figure 4 shows regions, where the AH effect is more probable than A with hollow circles (otherwise filled). There are no such regions for HDL-C showing even moderate signal for association and only few for LDL-C. The strongest region, with posterior association probability of 0.41, is in chromosome 11. SNPs in this region showing association are located in *LDLRAD3* gene.

Figure 5 shows also a comparison of the significance values from PLINK A and AH for HDL-C highlighting a clear qualitative difference in the behavior of BMA and single-SNP analysis. Moreover, only three of the seven regions with posterior association probability over half for HDL-C reach genome-wide significance level (here 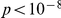) in the single-SNP analysis (Figure S7). For LDL-C, all of the five regions reach this level (except for one close call in AH analysis; Figure S7). PLINK AH indicates five SNPs for HDL-C with borderline genome-wide significance that are not picked up by only A model or the BMA models. These SNPs show clear recessive association patterns, but each with only a single observation having two minor alleles. It seems that testing the regression coefficients for statistical significance is not a very robust approach with additive and heterozygosity terms.

#### Heritability and the number of associated variants

**Figure 6:**
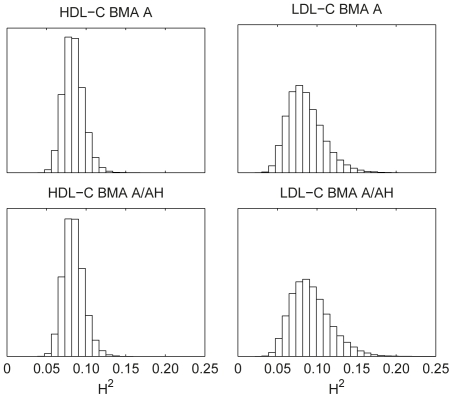
Posterior distributions of heritability for HDL-C and LDL-C. Median values are 0.08 for all except for LDL-C BMA A/AH, which has a median of 0.09.

Heritability samples were obtained from the MCMC as the proportion of (all phenotypic) variance explained by the included genetic effects (Figure 6). The median heritabilities for HDL-C were 0.08 with both methods and for LDL-C 0.08 and 0.09 with BMA A and A/AH, respectively. The histograms for HDL-C are nearly identical and are much narrower than those of LDL-C. LDL-C with BMA A/AH has slightly wider distribution and larger mean than with BMA A. Similar observations can be made for the number of included variants in the models: the medians [with 95% central posterior intervals] are 32 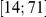 and 29 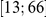 for HDL-C with BMA A and A/AH, and 42 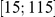 and 44 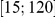 for LDL-C.

## Discussion

Our results demonstrate that Bayesian variable selection and model averaging (BMA) in searching for additive and dominant genetic effects is feasible on genome-wide scale and has several potential benefits over single-SNP analysis. The primary interest in genome-wide association studies (GWAS) often lies in locating causal variants (or variants linked to them), which may provide insight into the underlying biology of the phenotype, indicate new therapeutic targets for diseases and enable personalized risk analytics [1]. As complex traits are thought to involve multiple genetic effects, it is not surprising that the simultaneous analysis of all available variants has been found to increase the identification accuracy (at least in simulations) [3]–[6]. Our simulation study supports this (Figure 1). Our results also imply that if genetic dominance is present, allowing heterozygosity (AH) terms in the BMA models can provide a further increase in the accuracy. Moreover, in the analysis of HDL-C blood concentrations in 3985 Finns, BMA highlighted regions with previously reported associations, which did not reach genome-wide significance in the single-SNP analysis of this dataset. Further analysis of the implied associations is out of the scope of our article.

Notably, even if only additive effects were simulated, there was virtually no decrease in the identification accuracy from allowing AH terms. This behavior can (at least partly) be explained by allowing the data to provide information on the relative numbers of the different types of effects 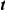 through their prior (Equation 2). The effect of the prior is also clearly visible when comparing the results of BMA and single-SNP analysis for the HDL-C data in Figure 5. In the current form, the model becomes more and more conservative against other effect types as more and more variants with a single effect type are added. In situations where this is undesirable, the prior could be fixed to uniform over the effect types or its strength relative to the number of included variants could be controlled (by allowing 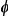 to depend on 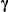). However, this behavior is a demonstration of the key feature of “sharing of strength” in hierarchical modeling, and highlights one of many potential benefits in the simultaneous analysis of all variants.

We also compared the BMA A/AH approach to a pseudo-SNP approach, which doubles the number of variants by introducing an additional pseudo SNP with heterozygosity coding for each original SNP. This allows simple modeling of dominance without requiring any special implementation handling different effect types. However, our simulations indicate that an explicit model for the dominance variation may increase the performance of identifying associations. Moreover, the prior specification of model size and effect types are more natural when an explicit model is used. The performance for the identification of the types of effects had mixed results in our simulations.

The BMA approach facilitates posterior inference on heritability based on genotype data of unrelated individuals as studied by Guan and Stephens [6]. Here it is useful to note the difference between narrow- and broad-sense heritability. The former is defined as the proportion of phenotypic variance explained by additive genetic effects, while the latter includes also non-additive components (e.g., dominance). Hill et al. [35] argue based on literature and theoretical considerations that the additive component is expected to account for most of the genetic variance of complex traits. Our simulation and real data results seem to be in line with this (disregarding the possibility of gene-gene and gene-environment interactions). Although in the second set of our simulations two thirds of the effects are either dominant or recessive, models with only additive effects, while being clearly biased downwards, seem to capture a large part of the overall heritability (Figure 2). For HDL-C and LDL-C, a large meta-analysis study reported that about 12% of the total variances of each were explained by the identified SNPs, which is only around 25 to 30% of the genetic variances of the traits [34], highlighting the general observation that associations in SNP data often account for a small part of total heritability [2]. The cited values are at the upper ends of our posterior distributions (Figure 6). Our results imply no dominance component for HDL-C and a possibility of a small dominance component for LDL-C.

A few issues regarding our modeling choices and computation should be highlighted. First, the distribution for the effect sizes was assumed normal for computational convenience, although a heavier tailed distribution could be more robust and often truer to prior assumptions in GWAS (see, e.g., Park et al. [36]). Double exponential distribution was used to generate the effect sizes in the simulations, which may explain the bias to small model sizes in the results (Figure 3). Yet, the inferences on heritability seem well-calibrated (Figure 2). However, the normal assumption may be an issue if there are some variants with large effects and lots of variants with small effects. Then, the strong associations will increase the variance of the effect size distribution (via 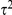), which will affect the implicit threshold in the spike-and-slab prior to include variants into the model.

Another issue concerns the computation. The local updating scheme of variant inclusion (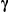) suffers from large autocorrelations and may perform poorly if the distribution is multi-modal. Indeed, there were indications of poor mixing between near-by SNPs, but the calculation of region-wise posterior association probabilities did not seem to suffer from this. A further analysis of the associated regions would need to take the potential problems into account. We also note that our specification of the regions based on the HapMap genetic maps is simplistic and intended for summarization (similarly, Guan and Stephens [6] divide the genome into regions based on basepair positions). That the single-SNP analysis for HDL-C and LDL-C did not indicate any significant associations not seen in the BMA results (apart from the few probable false positives addressed in the results), implies that there at least is no such multi-modality, which would mask strong associations. However, if multi-modality becomes a problem, incorporating global moves between tempered chains from the Evolutionary Monte Carlo of Bottolo and Richardson [37] to the current scheme could be of help, albeit with the cost of increased computational burden. With the settings described in the Results section, the computation of a single chain for BMA A/AH model took approximately 96 hours for the HDL-C and LDL-C datasets (utilizing one core of 2.6 GHz AMD Opteron 2435 CPU). Our implementation is memory-efficient and allows running multiple parallel chains, which share the same dataset.

## Supporting Information

Text S1 **Details of the sampling scheme.**
(PDF)

Text S2 **PLINK analysis options.**
(PDF)

Figure S1 **True positive rate as a function of false positive rate in simulations with all forty replicate datasets combined within each configuration.** sB refers to Bayesian single-SNP analysis. Regions were defined based on HapMap genetic maps with 0.005 cM cutoff. sB A and PLINK A may be difficult to distinguish because of overlap.
(TIF)

Figure S2 **True positive rate as a function of false positive rate in simulations with all forty replicate datasets combined within each configuration.** sB refers to Bayesian single-SNP analysis. Regions were defined using the default LD block algorithm in the Haploview software [26]. sB A and PLINK A may be difficult to distinguish because of overlap.
(TIF)

Figure S3 **True positive rate as a function of false positive rate in simulations with all forty replicate datasets combined within each configuration.** sB refers to Bayesian single-SNP analysis. Regions were defined based on HapMap genetic maps with 0.01 cM cutoff (this figure is the same as in the main article expect for the inclusion of the sB results). sB A and PLINK A may be difficult to distinguish because of overlap.
(TIF)

Figure S4 **Comparison of the posterior association probabilities between BMA A/AH MCMC chains for HDL-C and LDL-C (SNP-wise and region-wise with the regions from HapMap genetic maps with 0.01 cM cutoff).**
(TIF)

Figure S5
**Region-wise BMA A posterior association probabilities for ten permutations of the HDL-C data.** The trait 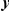 and the rows of the matrix 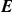 were randomly permuted (both with the same permutation), while the genotypes 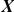 were left to the original order. The same hyperparameters and MCMC settings were used as with the original dataset.(TIF)

Figure S6 **Estimates of the number of associated variants in the regions (using HapMap genetic maps with 0.01 cM cutoff) for HDL-C and LDL-C with BMA A/AH.** Calculated for each region as a sum of the Rao-Blackwellized posterior association probabilities of the SNPs within the region [6].
(TIF)

Figure S7
**Region-wise BMA A/AH posterior association probabilities (gray) and PLINK AH** 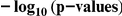 **(green; truncated at 12) for HDL-C and LDL-C.**
(TIF)
