# Supplementary material for: Bayesian Variable Selection in Searching for Additive and Dominant Effects in Genome-Wide Data
Source: PLoS One. 2012 Jan 3;7(1):e29115. doi: 10.1371/journal.pone.0029115 (PMC3250410; doi:10.1371/journal.pone.0029115)
Supplement: Text S1 — Details of the sampling scheme. (PDF) [file pone.0029115.s001.pdf]

Bayesian Variable Selection in Searching for Additive and Dominant Effects in Genome-wide Data

Supplementary Text S1

Tomi Peltola, Pekka Marttinen, Antti Jula, Veikko Salomaa, Markus Perola, and Aki Vehtari

Details of the sampling scheme

The conditional distributions for Gibbs sampling are derived below. Note that ω and $\boldsymbol{\phi}$ are marginalized out analytically and need not enter the computations. To sample from the posterior

$$p(\boldsymbol{\gamma}, \boldsymbol{t}, \boldsymbol{\beta}, \boldsymbol{\alpha}, \sigma^2, \boldsymbol{\tau^2}, \boldsymbol{X}|\boldsymbol{y}, \boldsymbol{X}_{obs}) \propto p(\boldsymbol{\gamma}, \boldsymbol{t})p(\boldsymbol{\beta}|\sigma^2, \boldsymbol{\tau^2}, \boldsymbol{\gamma}, \boldsymbol{t})p(\boldsymbol{\alpha}|\sigma^2)p(\sigma^2)p(\boldsymbol{\tau^2})p(\boldsymbol{X}|\boldsymbol{X}_{obs})p(\boldsymbol{y}|\boldsymbol{\beta}, \boldsymbol{\alpha}, \sigma^2, \boldsymbol{X})$$

the following Gibbs sampling scheme is used (with all steps conditional on observed data $\boldsymbol{y}$ and $\boldsymbol{X}_{obs}$):

1. $x_{ij}|\boldsymbol{X}_{-ij}, \boldsymbol{\gamma}, \boldsymbol{t}, \boldsymbol{\beta}, \boldsymbol{\alpha}, \sigma^2, \boldsymbol{\tau^2}$ for all i, j.
2. $\boldsymbol{\tau^2}|\boldsymbol{\gamma}, \boldsymbol{t}, \boldsymbol{\beta}, \boldsymbol{\alpha}, \sigma^2, \boldsymbol{X}$.
3. $\boldsymbol{\gamma}, \boldsymbol{t}|\boldsymbol{\tau^2}, \boldsymbol{X}$.
4. $\sigma^2|\boldsymbol{\gamma}, \boldsymbol{t}, \boldsymbol{\tau^2}, \boldsymbol{X}$.
5. $\boldsymbol{\beta}, \boldsymbol{\alpha}|\boldsymbol{\gamma}, \boldsymbol{t}, \sigma^2, \boldsymbol{\tau^2}, \boldsymbol{X}$.

Note that $p(\boldsymbol{\beta}|\sigma^2, \tau^2, \boldsymbol{\gamma}, \boldsymbol{t}) = p(\boldsymbol{\beta_\gamma}|\sigma^2, \boldsymbol{\tau^2})p(\boldsymbol{\beta_{-\gamma}}) = p(\boldsymbol{\beta_\gamma}|\sigma^2, \boldsymbol{\tau^2})$ or 0 if some $\beta_j \neq 0$ with $\gamma_j = 0$. Further, only variants with $\gamma_j = 1$ affect the likelihood: $p(\boldsymbol{y}|\boldsymbol{\beta}, \boldsymbol{\alpha}, \sigma^2, \boldsymbol{X}) = p(\boldsymbol{y}|\boldsymbol{\beta_\gamma}, \boldsymbol{\alpha}, \sigma^2, \boldsymbol{X})$.

Step 1

$$\begin{aligned}
p(x_{ij}|\ldots) &\propto p(x_{ij})p(\boldsymbol{y}|\boldsymbol{\beta}, \boldsymbol{\alpha}, \sigma^2, x_{ij}, \boldsymbol{X}_{-ij}) \\
&= \text{Categorical}(x_{ij}|\theta^0_{ij}, \theta^1_{ij}, \theta^2_{ij})p(\boldsymbol{y}|\boldsymbol{\beta}, \boldsymbol{\alpha}, \sigma^2, x_{ij}, \boldsymbol{X}_{-ij}) \\
p(x_{ij}|\ldots) &= \text{Categorical}(x_{ij}|\hat{\theta}^0_{ij}, \hat{\theta}^1_{ij}, \hat{\theta}^2_{ij}),
\end{aligned}$$

where $\hat{\theta}^k_{ij} \propto \theta^k_{ij}p(\boldsymbol{y}|\boldsymbol{\beta}, \boldsymbol{\alpha}, \sigma^2, x_{ij} = k, \boldsymbol{X}_{-ij})$ and $\sum_k \hat{\theta}^k_{ij} = 1$.

Step 2

Here, $\boldsymbol{\tau^2}$ has one component in BMA A (τ_A^2) and two in BMA A/AH (τ_A^2, τ_H^2). The components can be sampled independently with only the corresponding part of $\boldsymbol{\beta}$ affecting the sampling.

$$
\begin{aligned}
p(\tau_t^2|\ldots) &\propto p(\tau_t^2)p(\boldsymbol{\beta}_t|\sigma^2,\tau_t^2,\boldsymbol{\gamma},\boldsymbol{t}) \\
&\propto (\tau_t^2)^{-(\nu_{\tau_t^2}/2+1)}\exp(-\frac{1}{2\tau_t^2}\nu_{\tau_t^2}s_{\tau_t^2}^2)(2\pi\sigma^2\tau_t^2)^{-q/2}\exp(-\frac{1}{2\sigma^2\tau_t^2}\boldsymbol{\beta}_{t,\boldsymbol{\gamma}}^T\boldsymbol{\beta}_{t,\boldsymbol{\gamma}}) \\
&\propto (\tau_t^2)^{-((\nu_{\tau_t^2}+q)/2+1)}\exp(-\frac{1}{2\tau_t^2}(\nu_{\tau_t^2}s_{\tau_t^2}^2+\frac{1}{\sigma^2}\boldsymbol{\beta}_{t,\boldsymbol{\gamma}}^T\boldsymbol{\beta}_{t,\boldsymbol{\gamma}})) \\
p(\tau_t^2|\ldots) &= \text{Inv-}\chi^2(\tau_t^2|\nu_{\tau_t^2}+q,\frac{\nu_{\tau_t^2}s_{\tau_t^2}^2+\frac{1}{\sigma^2}\boldsymbol{\beta}_{t,\boldsymbol{\gamma}}^T\boldsymbol{\beta}_{t,\boldsymbol{\gamma}}}{\nu_{\tau_t^2}+q}).
\end{aligned}
$$

Step 3

A Metropolis-Hastings step is used to update $\boldsymbol{\gamma}$ and $\boldsymbol{t}$. Parameters $\boldsymbol{\beta},\boldsymbol{\alpha}$ and σ^2 are integrated out analytically at this step giving the following marginal likelihood:

$$
\begin{aligned}
p(\boldsymbol{y}|\boldsymbol{X},\boldsymbol{\tau^2},\gamma,\boldsymbol{t}) &= \int p(\boldsymbol{\beta}|\sigma^2,\boldsymbol{\tau^2},\gamma,\boldsymbol{t})p(\boldsymbol{\alpha}|\sigma^2)p(\sigma^2)p(\boldsymbol{y}|\boldsymbol{\beta},\boldsymbol{\alpha},\sigma^2,\boldsymbol{X})d\boldsymbol{\beta}d\boldsymbol{\alpha}d\sigma^2 \\
&= \pi^{-N/2}|\boldsymbol{\Sigma}|^{-1/2}|\boldsymbol{X}_{\boldsymbol{\gamma}}^T\boldsymbol{X}_{\boldsymbol{\gamma}}+\boldsymbol{\Sigma}^{-1}|^{-1/2}\frac{\Gamma((\nu+N)/2)}{\Gamma(\nu/2)}(\nu s^2)^{\nu/2}(\nu s^2+S_{yy})^{-(\nu+N)/2},
\end{aligned}
$$

where $S_{yy}=\boldsymbol{y}^T\boldsymbol{y}-\boldsymbol{y}^T\boldsymbol{X}_{\boldsymbol{\gamma}}(\boldsymbol{X}_{\boldsymbol{\gamma}}^T\boldsymbol{X}_{\boldsymbol{\gamma}}+\boldsymbol{\Sigma}^{-1})^{-1}\boldsymbol{X}_{\boldsymbol{\gamma}}^T\boldsymbol{y}$ and $\boldsymbol{\Sigma}$ is the prior covariance matrix of $\boldsymbol{\alpha}$ and $\boldsymbol{\beta}_{\boldsymbol{\gamma}}$.

Step 4

Note that $\boldsymbol{\beta}$ and $\boldsymbol{\alpha}$ are integrated out analytically at this step.

$$
\begin{aligned}
p(\sigma^2|\ldots) &\propto p(\sigma^2)\int p(\boldsymbol{\alpha}|\sigma^2)p(\boldsymbol{\beta}|\sigma^2,\boldsymbol{\tau^2},\boldsymbol{\gamma},\boldsymbol{t})p(\boldsymbol{y}|\boldsymbol{\beta},\boldsymbol{\alpha},\sigma^2,\boldsymbol{X})d\boldsymbol{\alpha}d\boldsymbol{\beta} \\
&\propto (\sigma^2)^{-(\nu/2+1)}\exp(-\frac{1}{2\sigma^2}\nu s^2)(\sigma^2)^{-N/2}\exp(-\frac{1}{2\sigma^2}S_{yy}) \\
&= (\sigma^2)^{-((\nu+N)/2+1)}\exp(-\frac{1}{2\sigma^2}(\nu s^2+S_{yy})) \\
p(\sigma^2|\ldots) &= \text{Inv-}\chi^2(\sigma^2|\nu+N,\frac{\nu s^2+S_{yy}}{\nu+N}).
\end{aligned}
$$

Step 5

Let $\boldsymbol{c} = [\boldsymbol{\alpha}^T \; \boldsymbol{\beta}_{\boldsymbol{\gamma}}^T]^T$ and $\boldsymbol{\Sigma}$ be the corresponding covariance matrix of the prior.

$$
\begin{aligned}
p(\boldsymbol{c}|\ldots) &\propto p(\boldsymbol{c}|\sigma^2, \boldsymbol{\tau^2}, \boldsymbol{\gamma}, \boldsymbol{t}) p(\boldsymbol{y}|\boldsymbol{c}, \sigma^2, \boldsymbol{X}) \\
&= |2\pi\boldsymbol{\Sigma}|^{-1/2} \exp(-\frac{1}{2\sigma^2}\boldsymbol{c}^T\boldsymbol{\Sigma}^{-1}\boldsymbol{c})(2\pi\sigma^2)^{-N/2} \exp(-\frac{1}{2\sigma^2}(\boldsymbol{y} - \boldsymbol{X_c c})^T(\boldsymbol{y} - \boldsymbol{X_c c})) \\
&\propto \exp(-\frac{1}{2\sigma^2}[(\boldsymbol{c} - (\boldsymbol{X_c}^T\boldsymbol{X_c} + \boldsymbol{\Sigma}^{-1})^{-1}\boldsymbol{X_c}^T\boldsymbol{y})^T(\boldsymbol{X_c}^T\boldsymbol{X_c} + \boldsymbol{\Sigma}^{-1})(\boldsymbol{c} - (\boldsymbol{X_c}^T\boldsymbol{X_c} + \boldsymbol{\Sigma}^{-1})^{-1}\boldsymbol{X_c}^T\boldsymbol{y})]) \\
p(\boldsymbol{c}|\ldots) &= \mathrm{N}(\boldsymbol{c}|(\boldsymbol{X_c}^T\boldsymbol{X_c} + \boldsymbol{\Sigma}^{-1})^{-1}\boldsymbol{X_c}^T\boldsymbol{y}, \sigma^2(\boldsymbol{X_c}^T\boldsymbol{X_c} + \boldsymbol{\Sigma}^{-1})^{-1}).
\end{aligned}
$$
