# Supplementary material for: Bayesian Variable Selection in Searching for Additive and Dominant Effects in Genome-Wide Data
Source: PLoS One. 2012 Jan 3;7(1):e29115. doi: 10.1371/journal.pone.0029115 (PMC3250410; doi:10.1371/journal.pone.0029115)
Supplement: Text S2 — PLINK analysis options. (PDF) [file pone.0029115.s002.pdf]

Bayesian Variable Selection in Searching for Additive and Dominant Effects in Genome-wide Data

Supplementary Text S2

Tomi Peltola, Pekka Marttinen, Antti Jula, Veikko Salomaa, Markus Perola, and Aki Vehtari

PLINK analysis options

PLINK version 1.07 [Purcell et al., 2007] was used for the single-SNP analyses. Results with additive genetic model (PLINK A) were obtained by running PLINK with `--linear` option and results with additive and heterozygosity terms were obtained with `--linear` and `--genotypic` options (2 degrees of freedom test). Same covariates were included as in the Bayesian models.

References

Purcell S, Neale B, Todd-Brown K, Thomas L, Ferreira MAR, Bender D, Maller J, Sklar P, de Bakker PIW, Daly MJ, et al. 2007. PLINK: A Tool Set for Whole-Genome Association and Population-Based Linkage Analyses. Am J Hum Genet 81:559–575.
