# Supplementary figures and images for: Bayesian Variable Selection in Searching for Additive and Dominant Effects in Genome-Wide Data
Source: PLoS One. 2012 Jan 3;7(1):e29115. doi: 10.1371/journal.pone.0029115 (PMC3250410; doi:10.1371/journal.pone.0029115)

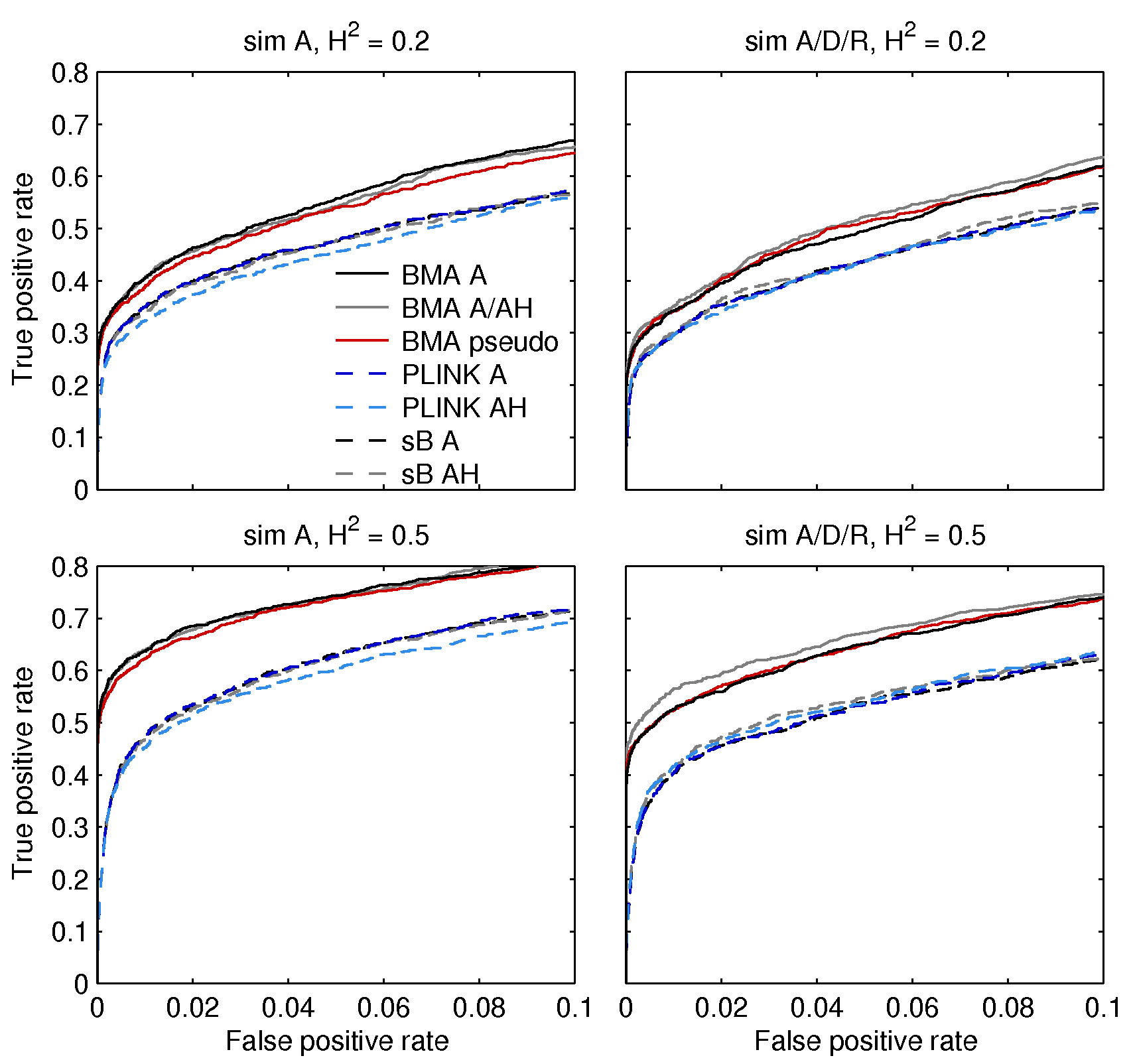

Supplement: Figure S1 — True positive rate as a function of false positive rate in simulations with all forty replicate datasets combined within each configuration. sB refers to Bayesian single-SNP analysis. Regions were defined based on HapMap genetic maps with 0.005 cM cutoff. sB A and PLINK A may be difficult to distinguish because of overlap. (TIF) [file pone.0029115.s003.tif]

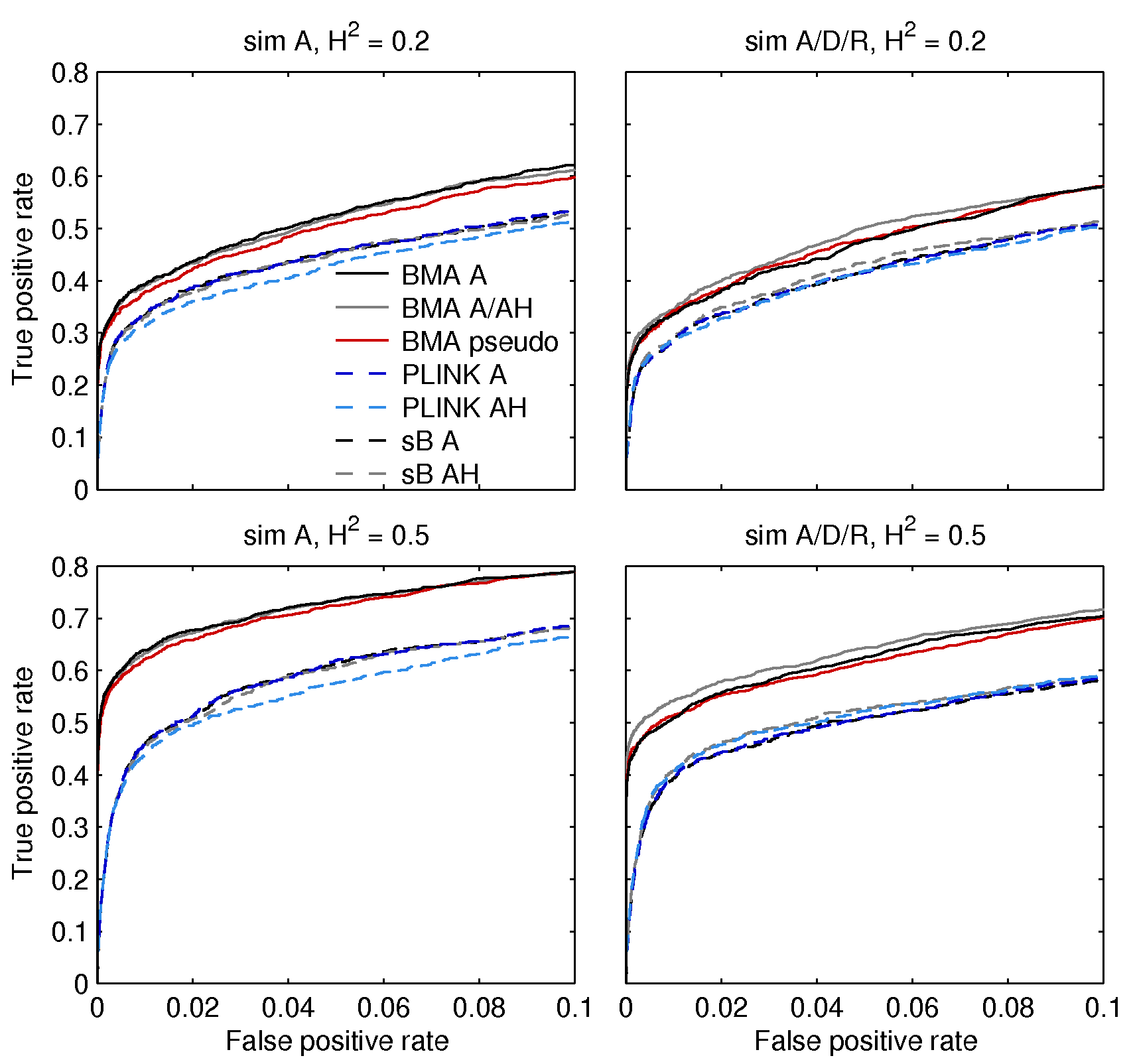

Supplement: Figure S2 — True positive rate as a function of false positive rate in simulations with all forty replicate datasets combined within each configuration. sB refers to Bayesian single-SNP analysis. Regions were defined using the default LD block algorithm in the Haploview software [26]. sB A and PLINK A may be difficult to distinguish because of overlap. (TIF) [file pone.0029115.s004.tif]

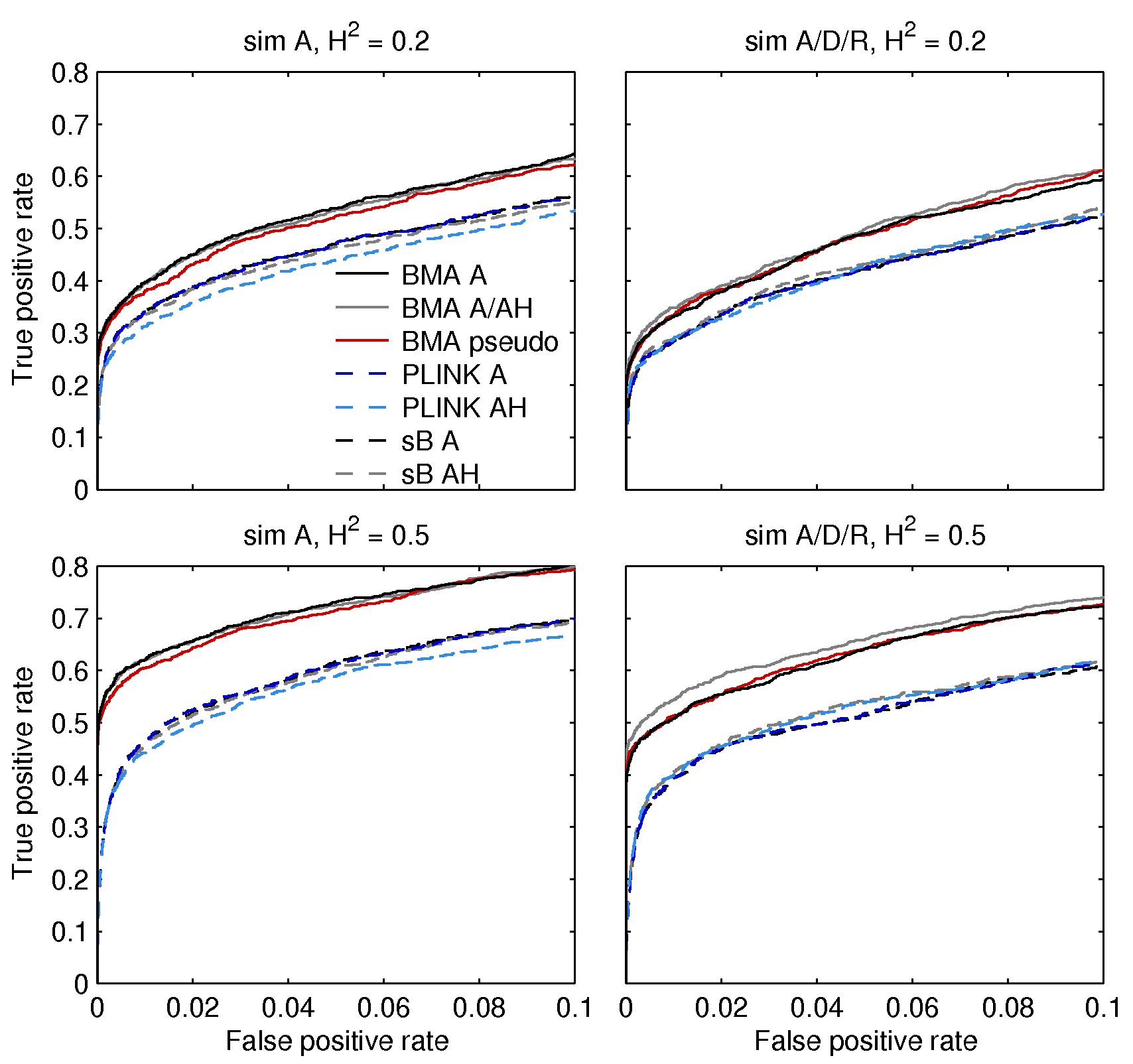

Supplement: Figure S3 — True positive rate as a function of false positive rate in simulations with all forty replicate datasets combined within each configuration. sB refers to Bayesian single-SNP analysis. Regions were defined based on HapMap genetic maps with 0.01 cM cutoff (this figure is the same as in the main article expect for the inclusion of the sB results). sB A and PLINK A may be difficult to distinguish because of overlap. (TIF) [file pone.0029115.s005.tif]

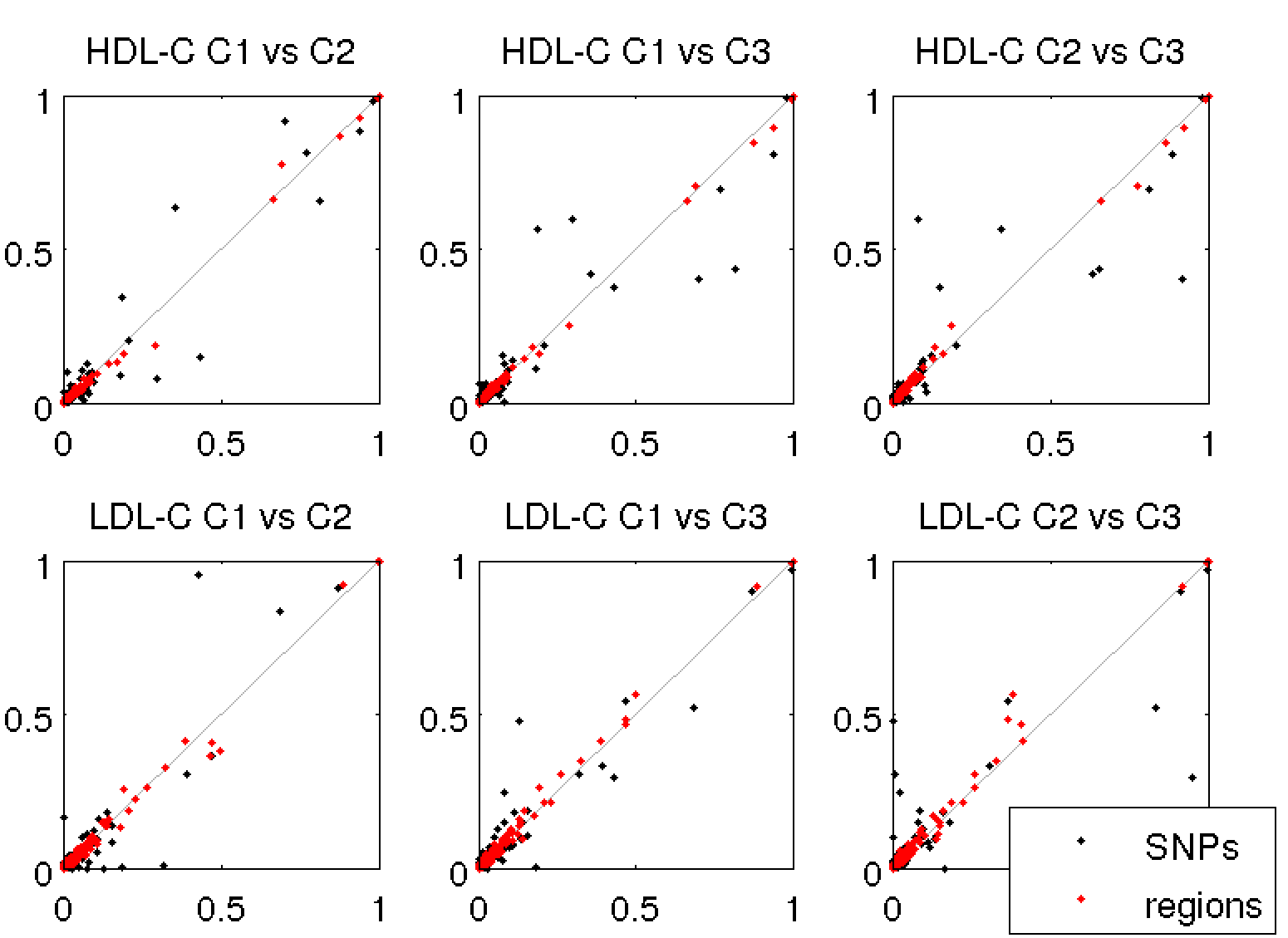

Supplement: Figure S4 — Comparison of the posterior association probabilities between BMA A/AH MCMC chains for HDL-C and LDL-C (SNP-wise and region-wise with the regions from HapMap genetic maps with 0.01 cM cutoff). (TIF) [file pone.0029115.s006.tif]

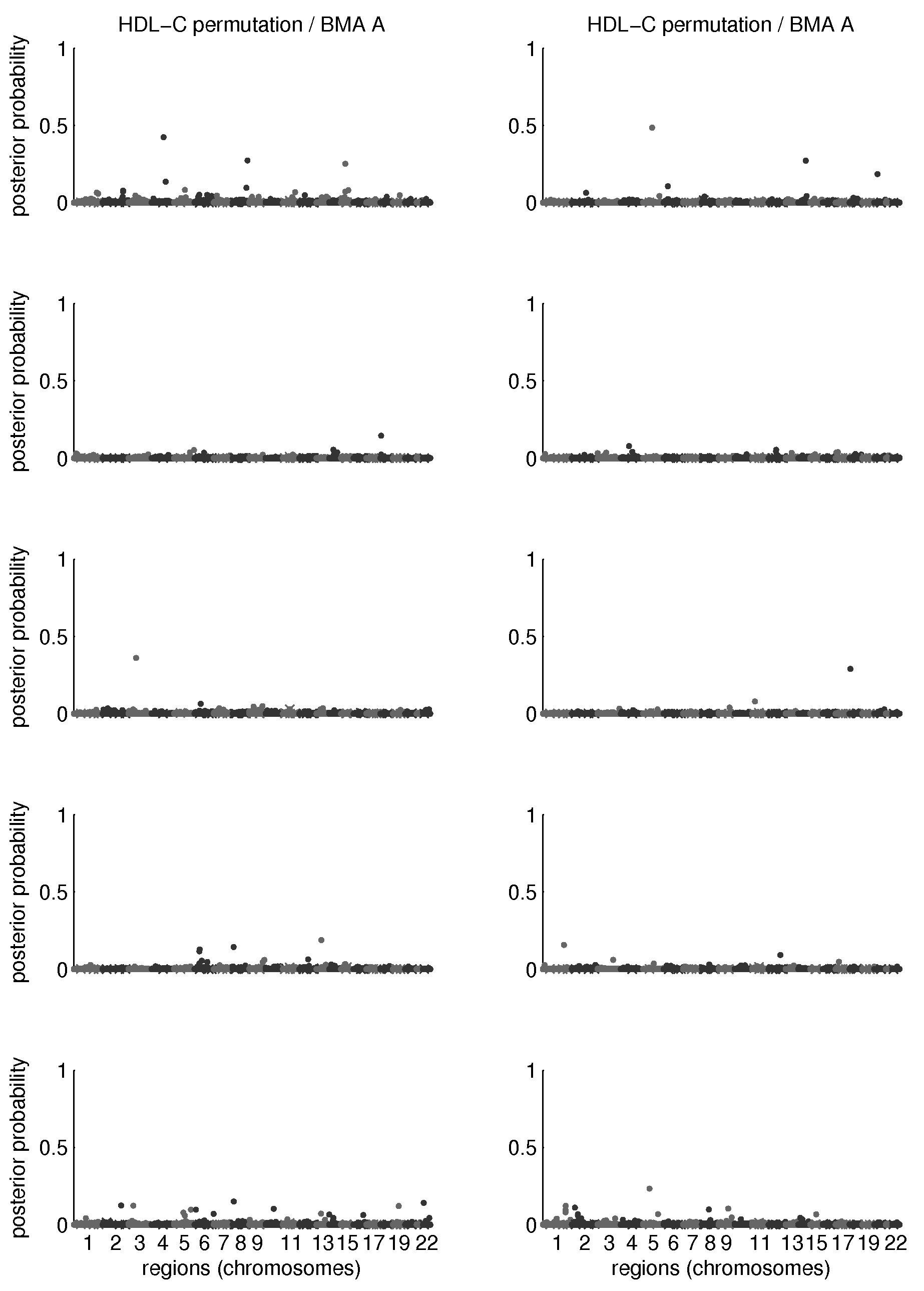

Supplement: Figure S5 — Region-wise BMA A posterior association probabilities for ten permutations of the HDL-C data. The trait and the rows of the matrix were randomly permuted (both with the same permutation), while the genotypes were left to the original order. The same hyperparameters and MCMC settings were used as with the original dataset. (TIF) [file pone.0029115.s007.tif]

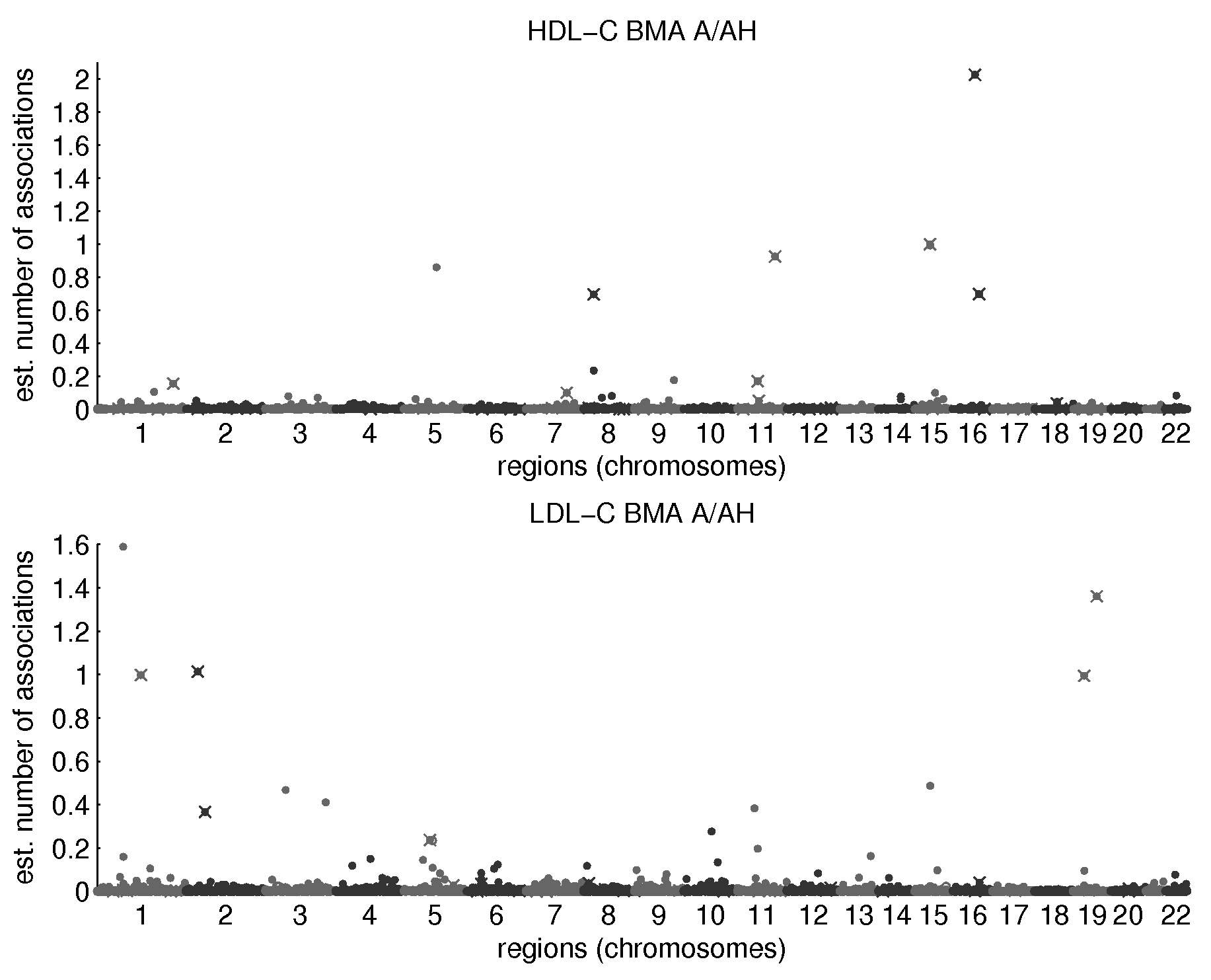

Supplement: Figure S6 — Estimates of the number of associated variants in the regions (using HapMap genetic maps with 0.01 cM cutoff) for HDL-C and LDL-C with BMA A/AH. Calculated for each region as a sum of the Rao-Blackwellized posterior association probabilities of the SNPs within the region [6]. (TIF) [file pone.0029115.s008.tif]

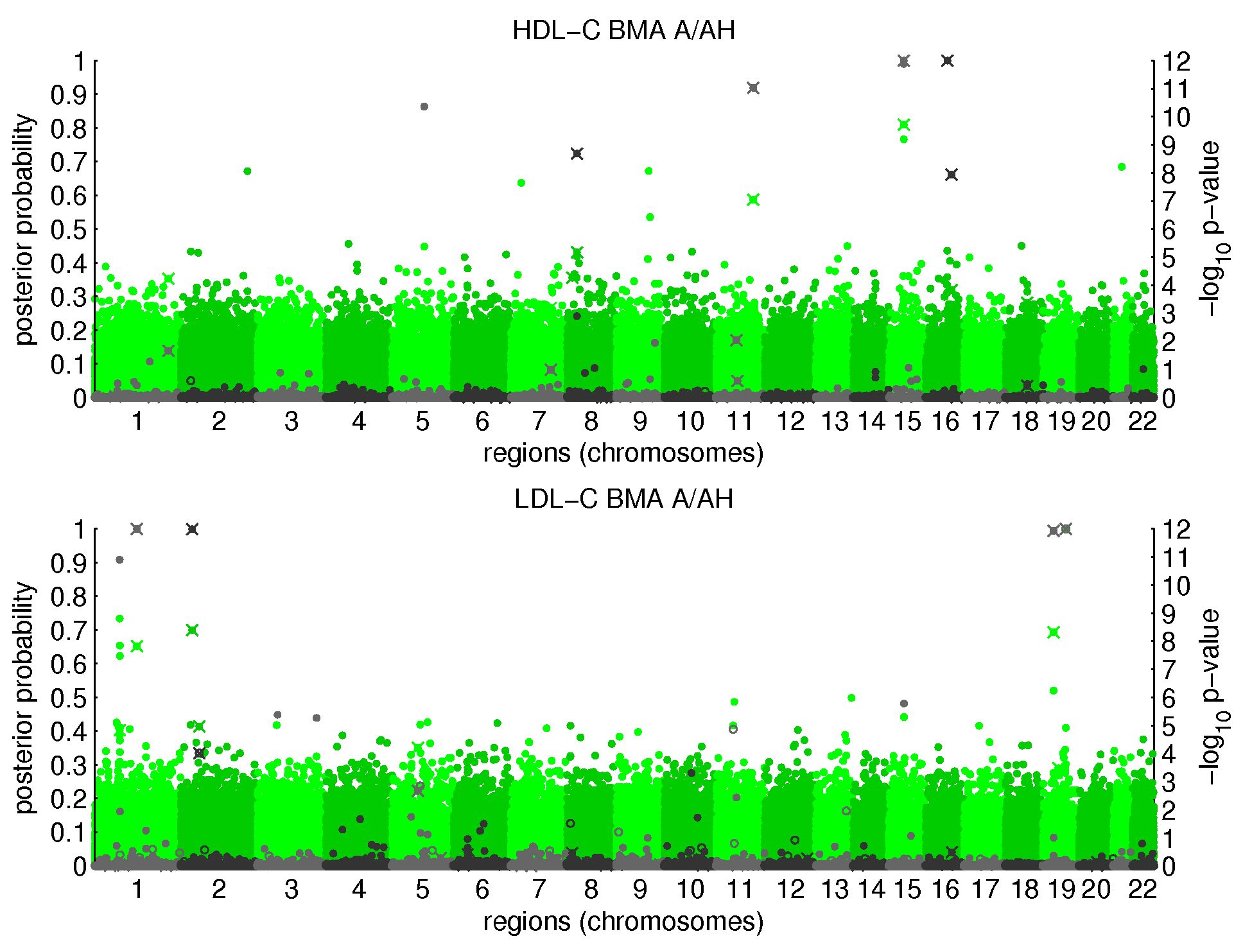

Supplement: Figure S7 — Region-wise BMA A/AH posterior association probabilities (gray) and PLINK AH (green; truncated at 12) for HDL-C and LDL-C. (TIF) [file pone.0029115.s009.tif]
